# A diagnostic miRNA signature for pulmonary arterial hypertension using a consensus machine learning approach

**DOI:** 10.1016/j.ebiom.2021.103444

**Published:** 2021-06-26

**Authors:** Niamh Errington, James Iremonger, Josephine A. Pickworth, Sokratis Kariotis, Christopher J. Rhodes, Alexander MK Rothman, Robin Condliffe, Charles A. Elliot, David G. Kiely, Luke S. Howard, John Wharton, A. A. Roger Thompson, Nicholas W Morrell, Martin R. Wilkins, Dennis Wang, Allan Lawrie

**Affiliations:** a Sheffield Institute for Translational Neuroscience, University of Sheffield, UK; b Department of Infection, Immunity & Cardiovascular Disease, University of Sheffield, Beech Hill Road, Sheffield, UK; c National Heart & Lung Institute, Imperial College London, Hammersmith Campus, Du Cane Road, London, UK; d Sheffield Pulmonary Vascular Disease Unit, Royal Hallamshire Hospital, Sheffield, UK; e National Pulmonary Hypertension Service, Imperial College Healthcare Trust NHS, Hammersmith Hospital, Du Cane Road, London, UK; f Department for Medicine, University of Cambridge, UK; g Department of Computer Science, University of Sheffield, UK; h Singapore Institute for Clinical Sciences, Singapore, Singapore

**Keywords:** Machine learning, Biomarkers, PAH, MicroRNA

## Abstract

**Background:**

Pulmonary arterial hypertension (PAH) is a rare but life shortening disease, the diagnosis of which is often delayed, and requires an invasive right heart catheterisation. Identifying diagnostic biomarkers may improve screening to identify patients at risk of PAH earlier and provide new insights into disease pathogenesis. MicroRNAs are small, non-coding molecules of RNA, previously shown to be dysregulated in PAH, and contribute to the disease process in animal models.

**Methods:**

Plasma from 64 treatment naïve patients with PAH and 43 disease and healthy controls were profiled for microRNA expression by Agilent Microarray. Following quality control and normalisation, the cohort was split into training and validation sets. Four separate machine learning feature selection methods were applied to the training set, along with a univariate analysis.

**Findings:**

20 microRNAs were identified as putative biomarkers by consensus feature selection from all four methods. Two microRNAs (miR-636 and miR-187-5p) were selected by all methods and used to predict PAH diagnosis with high accuracy. Integrating microRNA expression profiles with their associated target mRNA revealed 61 differentially expressed genes verified in two independent, publicly available PAH lung tissue data sets. Two of seven potentially novel gene targets were validated as differentially expressed *in vitro* in human pulmonary artery smooth muscle cells.

**Interpretation:**

This consensus of multiple machine learning approaches identified two miRNAs that were able to distinguish PAH from both disease and healthy controls. These circulating miRNA, and their target genes may provide insight into PAH pathogenesis and reveal novel regulators of disease and putative drug targets.

**Research in context**

**Evidence before this study**

Multiple reports exist on the expression and / or function of individual miRNAs in PAH, and reports of miRNA signatures in other disease but when we searched PubMed database using the terms [(“Pulmonary Arterial Hypertension” OR “PAH”) AND (“machine learning” OR “ensemble learning”) AND (“microRNA” OR “miRNA” OR “miR”)] for articles before February 20th 2021 and returned 0 results. PAH is a rare disease, and there is often a significant lag between symptom onset and patient diagnosis. Current clinically used blood based biomarkers are limited to markers of cardiac stress e.g. NT-proBNP that gives little insight into early pulmonary vascular disease, or the molecular drivers of disease. We hypothesised applying machine learning to microRNAs in PAH may provide novel insights.

**Added value of this study**

**This is the largest microRNA profiling of PAH patients with** 64 treatment naïve patients (sampled at the time of diagnosis), and 43 disease and healthy controls. It is also the first machine learning assessment of microRNAs for PAH.

**Implications of all the available evidence**

Our findings extend preliminary evidence that microRNAs may be able to classify PAH patients from controls, and suggest that a machine learning approach may allow for the detection of novel disease regulators.

Alt-text: Unlabelled box

## 1 Introduction

Pulmonary arterial hypertension (PAH) is a rare but progressive cardiopulmonary disease characterised by increased pulmonary vascular resistance driven by a sustained pulmonary arterial vasoconstriction and pulmonary vascular remodelling that leads to right heart failure and premature death. PAH pathogenesis is progressive and includes vasoconstriction, endothelial cell dysfunction, vascular cell proliferation and recruitment of circulating inflammatory cells. PAH can be further sub-categorised into seven sub-groups: Idiopathic PAH (IPAH), heritable PAH (HPAH), drug and toxin induced, PAH associated with other associated diseases, PAH long term responders to calcium channel blockers, PAH with overt features of venous/capillary involvement, and persistent PH of the newborn [1]. The molecular mechanisms of PAH are complex and include the influence of common [2] and rare genetic variation [3], epigenetic dysregulation of DNA methylation state, histone acetylation and microRNA (miRNA) dysregulation [4].

Often insidious at onset, PAH is usually rapidly progressive and patients frequently experience significant delays between initial symptom onset, diagnosis (right heart catheter) and treatment, with little improvement to these delays over that past 20 years [5,6]. Screening for PAH in connective tissue diseases (CTDs), including systemic sclerosis (SSc) where up to 10-15% of patients develop PAH has been shown to be beneficial [7] with several screening tools now available (reviewed in [5] recommended [8]). Screening for other forms of PAH is required, and the identification of blood-based biomarkers may help identify patients at risk earlier and reveal drivers of disease [5,9]. Current clinically used blood based biomarkers are limited to markers of cardiac stress e.g. N-terminal pro B-type Natriuretic Peptide (NT-proBNP) that gives little insight into early disease, or the molecular drivers of disease.

MicroRNAs (miRNA) are small, non-coding RNA molecules found in tissues, blood and plasma. They have been shown to be dysregulated in PAH, and contribute to the disease process in animal models [[10], [11], [12]]. Blood based miRNA biomarkers can be collected without the need for invasive tissue biopsy, and are present in plasma and serum in a stable form. However, with as many as 2300 miRNAs regulating biological processes [13], identifying those relevant for diagnosis of PAH can be computationally challenging.

Machine learning as a field has progressively improved our ability to find relevant features in large and high-dimensional data sets collected from genomic studies [14]. Supervised machine learning methods have been used successfully to develop classifiers for disease diagnosis, as well as to identify potential disease biomarkers [15]. Specifically in PAH we have previously utilised machine learning approaches to study molecular drivers of, and biomarkers for PAH [9,[16], [17], [18]]. In this study, we identify miRNA biomarkers associated with PAH selected using a consensus of four different supervised machine learning feature selection techniques. We assess the potential of miRNAs as a diagnostic tool by creating binary predictive classification models, and assessing the accuracy of these models. Further insight into the role of miRNAs in the pathogenesis PAH and potential candidates for therapeutic intervention is revealed through the analysis of miRNA target genes and pathways in human lung and whole blood transcriptomes.

## 2 Methods

### 2.1 Cohort overview and sample collection

We collected 83 unique plasma samples from sequentially consented patients with suspected pulmonary hypertension and controls, obtained according to the Declaration of Helsinki, with local research ethics committee approval and informed written consent from all subjects from the Sheffield Teaching Hospitals Observational study into Pulmonary Hypertension, Cardiovascular and Lung disease Biobank (STH-Obs, UK REC 18/YH/0441). Patient samples were obtained from the diagnostic right heart catheter and were PAH-treatment naïve. From the 83 samples, 18 patients with SSc-associated PAH (SSc-PAH) and 10 SSc patients without PH (SSc-without PH) were incorporated into the PAH patient groups and controls respectively. All patients with SSc were of the limited cutaneous subtype. The rest of the Sheffield samples were comprised of 34 IPAH patients and 21 healthy controls. An additional 24 patient and healthy control samples were obtained from the Imperial College London Pulmonary Hypertension sample collection (UK REC 17/LO/0563) and included in the study to remove a single centre bias. All samples were collected between 2007 and 2013, then stored in plasma at -80oC until the miRNA extraction. The cohort comprising all available samples meeting these criteria at the time of miRNA extraction, was randomly assigned to training (two-thirds) and validation (one-third) sets, matched for age, sex and WHO functional class, with demographics seen in Table 1. The training set was used to build models, which were evaluated in the validation set to minimise overfitting bias. Principal component analysis showing the clustering of patients can be found in Supplementary Figure 1.

**Table 1:** Basic demographics for a cohort of healthy controls (HC) and patients with PAH from Sheffield and Imperial, profiled for miRNA expression. Patients with systemic sclerosis (SSc) included in both the HC and PAH classification sets. Not all metrics available for all patients. For missing values, see Supplementary table 1.

	**Training Set**		**Validation Set**	
	**HC + SSc without PH**	**IPAH + SSc-PAH**	**HC + SSc without PH**	**IPAH + SSc-PAH**
No. Sheffield samples	14 + 7	23 + 11	7 + 3	11 + 7
No. Imperial Samples	8 + 0	8 + 0	4 + 0	4 + 0
**Total sample no.**	**29**	**42**	**14**	**22**
Mean age at sampling (years)	54.1 (14.5)	56.5 (14.3)	51.6 (11.7)	57.4 (15.3)
Female (%)	12 + 6 (58.1%)	18 + 6 (57.1%)	7 + 3 (71.4%)	8 + 6 (63.6%)
Alive 5 years follow up (%)	28 (97%)	28 (65%)	14 (100%)	9 (43%)
WHO Functional class (1,2,3,4)	-	(0,6,33,3)	-	(0,3,17,2)
Patients on immunomodulatory agent at sampling	4	2	2	2
Mean Pulmonary Arterial Pressure (mm Hg)		54.9 (15.6)		49.4 (13.7)
Pulmonary vascular resistance (dynes)		870 (488)		753 (448)
6 minute walk distance: Imperial only (m)		202 (158)		378 (59)
ISWD: Sheffield only (m)		214 (169)		248 (246)
Cardiac Output (L/min)		4.8 (1.4)		5.0 (2.0)
Mean pulmonary arterial wedge pressure (mm Hg)		10.4 (3.8)		10.8 (3.2)

Continuous variables described as mean (standard deviation)

#### 2.1.1 Plasma preparation and RNA isolation

Total RNA was isolated from 1 ml of Citrate plasma using the Norgen total RNA slurry format extraction kit (Norgen Biotek Corp. Canada). RNA was concentrated using the RNA Clean and Concentrate-5 kit (Zymo Research Corp, U.S.A). Detailed methods can be found in the Supplementary materials.

#### 2.1.2 Microarray profiling and preprocessing

**Fig. 1.**
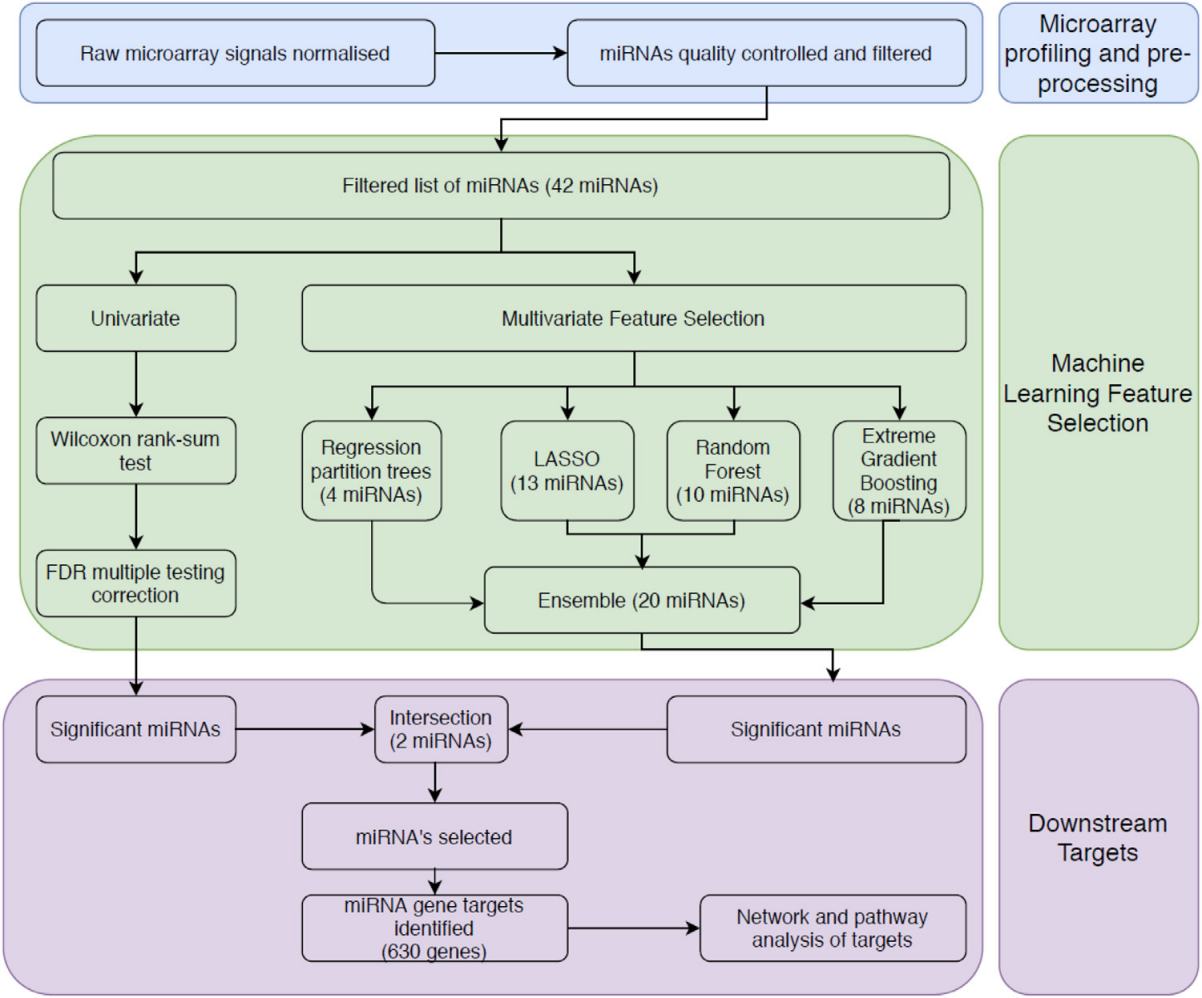
Machine learning methodology for the identification of miRNAs which may play a role in PAH, and the assessment of their target genes.

Agilent single colour miRNA arrays miRbase v.19 (Agilent Technologies, UK), which can detect up to 2006 human miRNAs, were performed on purified and concentrated plasma RNA in 2015. Raw microarray signals were normalised using the quantile method within the robust mean array (RMA) method from the R package AgiMicrorna (v.2.14.0) [19], correcting for the background signal. MiRNAs were then filtered, keeping only those expressed in at least 10% of arrays, leaving 393 miRNAs. Expression levels were log2 transformed and all subsequent calculations were performed on this value. MiRNAs were filtered down to 179 by those which have been qPCR confirmed to exist by Exiqon, and therefore, we can assume they can be accurately quantified by the Agilent array. We further eliminated features with high mean absolute correlation, using a correlation matrix method. For each feature, the mean absolute correlation based on pair-wise correlations was calculated. If a pair-wise correlation was > 0.7, the feature with the greater mean absolute correlation was removed, using the caret package (v6.0-86) in R. Where two miRNAs are highly correlated both with each other and disease status, and both are kept in the model, there is a danger that both may be considered insignificant, potentially missing an important signal. We carried forward our downstream analysis with 42 miRNAs after filtering. The workflow is described in Fig. 1.

We used PCA and t-SNE analysis to visibly explore the data. PCA analysis was carried out using prcomp in R without scaling the data, and a t-SNE analysis was run using the Rtsne package.

### 2.2 Statistical analysis

#### 2.2.1 Multivariable microRNA selection and model building

All statistical analyses were carried out using R (v4.0.0) [20]. We used both a multivariable and univariable approach to selecting miRNAs. In the multivariable approach, we used four separate feature selection methods simultaneously to identify candidate biomarkers, with the intersection amongst the methods considered the significant miRNAs. In each instance, parameters were tuned using 10-fold cross-validation (repeated 10 times) on the training set. For each of the feature selection methods, we subsequently used a supervised machine learning approach for binary classification to create predictive classification models, based on features selected from the prospective cohort study. For further details on the parameters used, see the code available on github at https://github.com/niamherrington/microarray-miRNA. The guidelines of the transparent reporting of a multivariable prediction model for individual prognosis or diagnosis (TRIPOD) statement were followed (Supplementary Table 2).

##### Random forest using Boruta

Boruta is a feature selection random forest wrapper algorithm designed to identify all relevant variables in a classification framework [21]. We performed 300 iterations of the random forest normalised permutation importance function to obtain attribute importance, using default settings within Boruta package (v7.0.0) in R, including the confidence level of 0.01. After the 300 runs were complete, miRNAs still not confidently classified as important variables were rejected along with the miRNAs rejected by the algorithm. This process was then repeated 100 times, with miRNAs selected on at least 10 occasions were carried forward.

We then combined the microRNAs selected by Boruta into a random forest model using the randomForest package (v.4.6-14) [22]. We selected a random forest model as they are generally robust to overfitting, and capable of learning non-linear relationships. However, the results may not be easily interpretable. The caret package was used to identify 1000 trees as being optimal among the 100, 250, 500, 750, 1000, 1250 and 1500 trees tested. The number of variables available for splitting at each tree node was optimised next, with 1 variable per tree node the best out of a range from 1 to 4. A probability threshold of > 0.5 was used to determine whether a subject was a PAH patient or no PH.

##### Regression partition tree

Classification trees were calculated using Rpart (v4.1-15) [23] and caret in R. A major advantage of rpart is the interpretable output, that can be displayed graphically. However, a disadvantage is that the trees tend to have a lower predictive accuracy, due to the fact the trees are less robust. The trees were used by the greedy feature selection algorithm, recursive binary splitting to return ordered features, from the root of the tree down.

The fit of the model was controlled by setting the minimum number of observations that must exist in a node for a split to be attempted to four, and the minimum number of observations in any terminal node set to two. The trees were split by minimising the Gini index at each split. This was then cross-validated using 10-fold, repeated cross-validation. We considered a variable selected if it was present in the final tree. A probability threshold of > 0.5 was used to determine whether a subject was a PAH patient or no PH.

##### LASSO

Least absolute shrinkage and selection operator (LASSO) on binomial logistic regression using the glmnet package in R (v4.0) [24] was used to select relevant miRNAs, by eliminating parameters with a coefficient of 0. One of the advantages to using a LASSO method is that coefficients are shrunk and removed, reducing variance without substantially increasing the bias [25]. Additionally, LASSO models allow for effectively interpretable output. However, a drawback to LASSO is a lack of flexibility to fully capture non-linear relationships. We chose the regularisation parameter, λ, using 10-fold cross-validation with binomial deviance as the criterion. From the cross validations, the value of λ with the minimum binomial deviance (λ-min = 0.0502) was selected and used to refit the model. A probability threshold of > 0.5 was used to determine whether a subject was a PAH patient or no PH. To ensure the models were not driven by age and sex, we also attempted to classify patients using these characteristics in a LASSO model.

##### XGBoost

The final model we used to fit miRNA features to disease diagnosis was the gradient boosting method, using the XGBoost package in R (v1.0.0.1) [26]. We trialled XGBoost as it has been used very effectively in a range of classification problems, consistently winning machine learning competitions on Kaggle, as well as providing insights into biological data sets. However, with many hyperparameters to tune, computational time is longer than some of the other methods, additionally, the results can be difficult to interpret. XGBoost is an extreme gradient boosting method which ranks the features from most to least important. To decide on the regularisation parameter settings, we used a grid search over a range of values, using 10-fold repeated cross-validation on the training set, selecting the optimal values for the final model (Supplementary Table 3). The optimisation ranges were selected by expanding grid searches previously used by other teams on RNAseq data [27]. The ability to fine-tune these parameters in XGBoost means the model is more robust to overfitting. Features contributing to more than a 5% improvement in accuracy to their branches were selected as ‘important’. A probability threshold of > 0.5 was used to determine whether a subject was a PAH patient or no PH. Once features had been selected, the model was retrained over the same parameter range, using just selected miRNAs.

##### Ensemble

An ensemble of predictions from the above classifiers were generated by averaging the predicted probabilities from each individual supervised machine learning approach, and then using a threshold of > 0.5 to call subjects with PAH.

##### Comparison with NT-proBNP

All patients, and healthy controls from Sheffield had routine clinical measurements of NT-proBNP. This information was used to compare the accuracy of the miRNA models with NT-proBNP as a classifier by retraining each of the models with NT-proBNP as an additional variable. The performance of standalone NT-proBNP for the cohort was also measured.

##### Multivariable classifier performance assessment

We also used a leave-one-out cross validation approach (LOOCV) to compare miRNAs selected when the entire dataset was used. All methods above were attempted across the whole dataset, using a LOOCV approach instead of repeated cross validations. AUCs were calculated using the average of the cross validations across the whole dataset, rather than using training and validation sets.

##### Classification without SSc

Finally, we repeated the above machine learning methods to classify patients with IPAH or healthy controls, using the same training and validation sets described above, without patients with SSc.

#### 2.2.2 Univariable analysis

Using a Shapiro-Wilk test [28] for the selected miRNAs, a normality assumption for the majority of miRNAs is violated. As a result, for each miRNA, we performed a non-parametric Wilcoxon rank-sum test, comparing expression levels between patients with PAH and the no PH group, to find a single p-value for each miRNA. These p-values were then adjusted using the Benjamini Hochberg multiple testing correction to control the false discovery rate (FDR) with a cutoff of 0.05. We calculated the discriminatory power of each individual miRNA, using the training set to find an optimal cutpoint by simultaneously maximising sensitivity and specificity, then calculating the accuracy using the validation set. We examined survival using the Kaplan-Meier method for each selected miRNA and calculated the p-value for a log-rank test. All participants were followed up for 5 years after the sample date, or date of death, with no participants lost to follow up. Cox proportional hazard tests were done using the survival package (v2.44-1.1)

#### 2.2.3 Combining MicroRNAs

To compare classifiers, we looked at how accurately each classifier categorised each patient in the validation set. We also looked at the performance of each feature selection method, by comparing them using the following evaluation metrics, where TP represents true positive, FN represents false negative, TN represents true negative, and FP represents false positive.

• Sensitivity = TP / (TP + FN)
• Specificity = TN / (TN + FP)
• Positive predictive value = TP / (TP + FP)
• Negative predicted value = TN / (TN + FN)
• Correct classification rate =(TP + TN) / (TP + TN + FP + FN)
• Area under the receiver operator characteristic (ROC) curve (AUC); the confidence interval calculated using the method by Delong et al [29].

### 2.3 Pathway analysis

Gene targets were inferred using DIANA v5.0 microT-CDS [30] for the miRNAs which appeared in all four features selection methods, with the threshold for target prediction set to the default of 0.7. We then carried out a network analysis using WebGestalt [31] and Cytoscape (v3.7.1) [32]. Pathway genes were downloaded from KEGG [33].

### 2.4 External validation in whole blood RNA seq

RNA sequencing was performed on whole-blood samples from 359 patients with PAH, and 72 volunteers, as previously described [34]. 28 of the Sheffield samples, and 2 Imperial healthy controls were also included in the miRNA cohort, so we excluded these to ensure the validation set was independent. We split the cohort into the same training and validation groups, and then used XGBoost to classify patients using the gene targets identified using similar optimisation ranges as above. As this dataset is unbalanced due to a comparatively small number of healthy controls, we incorporated a weighting parameter; number of PAH cases / number of controls. The final parameters selected can be seen in Supplementary Table 4. The threshold value was calculated using Youden's Index.

### 2.5 External validation in published lung tissue microarray studies

Two publicly available datasets profiling lung tissue from patients with PAH were used to validate the gene target lists. In GEO accession GSE15197 [35], differential expression was measured in 13 normal lung tissue samples compared to 18 lung tissue samples with PAH. We excluded seven samples where patients had PH secondary to idiopathic pulmonary fibrosis (IPF). The original study found 13,899 genes differentially expressed between patients with PAH and healthy controls. GEO accession GSE53408 [36] compared 12 samples of lung tissue from patients with PAH to 11 healthy lung tissue samples. Basic characteristics of the two cohorts are described in Supplementary Table 5.

The GEOR2 interface was used to import data into R using Biobase (v2.42.0) and GEOquery (v2.50.5). The limma package (v3.38.3) used for differential expression analysis with a log2 transform. Gene targets were extracted and FDR corrected (<0.05) using the Benjamini Hochberg correction.

### 2.6 qPCR validation of gene targets

Pulmonary artery smooth muscle cells (PASMCs) purchased from commercial suppliers (Lonza catalogue # CC-2581) taken from healthy donors and PASMCs isolated from four separate IPAH patients (donated from Prof. N Morrell of Cambridge University) as previously described [37], were grown in culture before being quiesced (0.2% foetal Calf Serum) for 48 hours, and lysed for the isolation of RNA using Trizol. Direct-zol RNA mini-prep kits (Zymo research R2050), and Zymospin column were used to extract RNA as per manufacturer's instructions. RNA (n=3 for each condition) was reverse transcribed to cDNA using RNA to cDNA kit (Applied Biosystems 4387406). Eight genes were selected for quantitative-PCR (qPCR) and TaqMan probes for FER (Hs00245497_m1), UCR3 (Hs00419575_m1), MTUS1 (Hs00368183_m1), API5 (Hs00362482_m1), PELI1 (Hs00900505_m1), HGF (Hs00300159_m1), GLMN (Hs00369634_m1), PARP8 (Hs01065404_m1) were purchased from Thermo Fisher and run in duplicate. Human ATP5B Hs00969569_m1was used as control. Relative quantity was calculated using the ΔΔCt method. Analysis was performed using GraphPad Prism v 8.2.

### 2.7 Role of funding source

The funders had no role in study design, data collection, data analyses, interpretation or writing of the report.

## 3 Results

We profiled the miRNAs from 64 patients with PAH and 43 combined SSc-without PH and healthy controls (no PH). Initial t-Distributed Stochastic Neighbour Embedding (t-SNE) and principal component analysis (Supplementary Figure 1) showed some separation between groups. Since several of the feature selection methods utilised later cannot account for multicollinearity, we undertook two filtration steps to reduce the starting number of miRNAs. Initially the miRNAs were filtered, removing those failing quality control, and miRNAs highly correlated to each other, to leave 42 miRNAs (Supplementary Figure 2). Next, we selected the miRNAs most predictive of PAH vs no PH using four different supervised machine learning methods.

### 3.1 miRNAs selected using supervised machine learning approaches

**Fig. 2.**
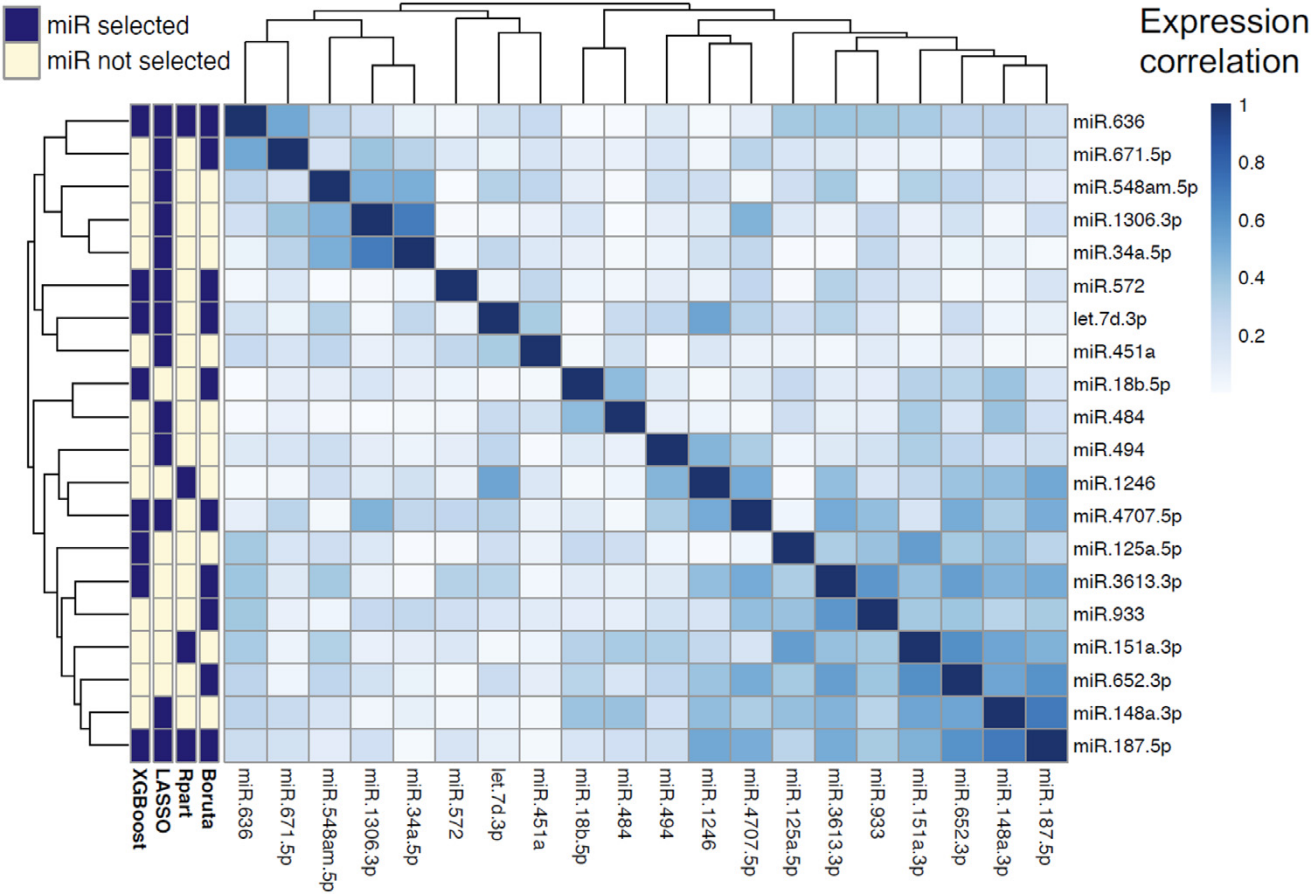
Expression correlation (Spearman) matrix between miRNAs selected by machine learning methods (side-bar). Dendrogram orders miRNAs by hierarchical clustering. XGBoost: Extreme gradient boosting method. Rpart: a regression partition tree method. Boruta: a random forest wrapper method for feature selection.

The disease diagnosis (PAH vs no PH) of 72 individuals was described as a function of the 42 miRNAs using four different machine learning methods. Feature selection was used to determine the miRNAs most relevant to the diagnosis. Four different machine learning techniques were used to select miRNAs and model PAH diagnosis; Boruta (an embedded random forest method), LASSO, regression partition trees, and XGBoost (an extreme gradient boosting method). The features subsets selected by each method were all different, though there were overlapping miRNAs in all (Fig. 2). Two miRNAs were selected by all four methods; miR-636 and miR-187-5p. These 2 miRNAs were the most consistently selected when different discovery sets were utilised; a training and validation set approach, leave-one-out cross validated approach, and a training and validation set approach without patients with SSc (Supplementary Figure 4).

### 3.2 Performance of PAH classification using miRNAs

To compare the performance of each feature selection method, we looked at how each model performed as a classifier on the validation set. The classification of each subject by each model can be seen in Supplementary Table 6. Boruta random forest had the highest overall accuracy, with 30 out of 35 subjects in the validation set correctly identified.

**Fig. 3.**
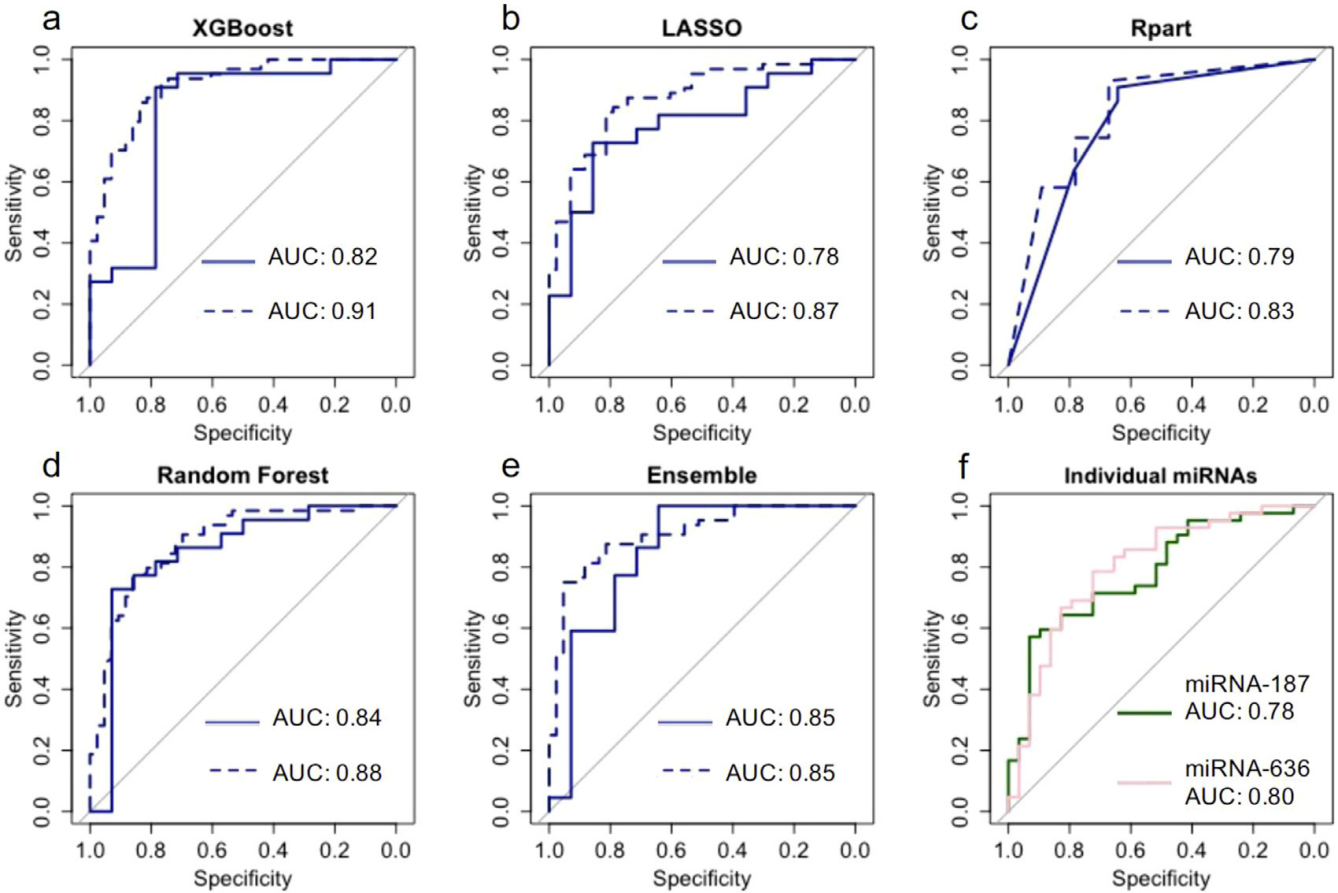
Solid lines indicate ROC for the validation set (n = 35), where the model was trained on a separate set. Dashed lines indicate miRNA models trained using a leave-one-out cross validation approach across the whole data set. (a) extreme gradient boosting (XGBoost) utilising 8 miRNAs; (b) LASSO utilising 13 miRNAs; (c) regression partition trees (Rpart) utilising 4 miRNAs; (d) a random forest wrapper method (Boruta) utilising 10 miRNAs; (e) Ensemble approach utilising 20 miRNAs (f) Average cross validated ROC for miRNA-187-5p and miRNA-636 on the training set.

The performance of each feature selection method on the validation set was also variable (Table 2). The cross validated performance for the training set can be seen in Supplementary Table 7. The Random Forest model had the highest AUC (0.84), but the XGBoost model had a higher accuracy (0.83). The LASSO model had the poorest performance, with an accuracy of 0.72. The number of miRNAs selected by each method also differed, with LASSO selecting the most (13 miRNAs), and the Rpart model behaving more stringently by selecting just four miRNAs. The AUCs for models trained using a leave-one-out cross-validation approach showed similar results (Fig. 3).

**Table 2:** Model performance of four classifiers on the validation set; a random forest wrapper method (Boruta), regression partition trees (Rpart), LASSO, and extreme gradient boosting (XGBoost).

	**Random forest**	**Rpart**	**LASSO**	**XGBoost**	**Ensemble**
miRNAs selected by model, n	10	4	13	8	20
Sensitivity (95% CI)	0.86 (0.65-0.97)	0.91 (0.71-0.99)	0.77 (0.55-0.92)	0.91 (0.71-0.99)	0.91 (0.71-0.99)
Specificity (95% CI)	0.71(0.42-0.92)	0.64 (0.35-0.87)	0.64 (0.35-0.87)	0.71 (0.42-0.92)	0.64 (0.35-0.87)
Positive predictive value (95% CI)	0.83 (0.61-0.95)	0.80 (0.59-0.93)	0.77 (0.55-0.92)	0.83 (0.63-0.95)	0.80 (0.59-0.93)
Negative predictive value (95% CI)	0.77 (0.46-0.95)	0.82 (0.48-0.92)	0.64 (0.35-0.86)	0.83 (0.52-0.98)	0.82 (0.48-0.92)
Correct classification rate (95% CI)	0.81 (0.64-0.92)	0.81 (0.64-0.92)	0.72 (0.55-0.86)	0.83 (0.67-0.94)	0.81 (0.64-0.92)
AUC (95% CI)	0.84 (0.69-1)	0.79 (0.63-0.95)	0.79 (0.63-0.94)	0.82 (0.66-0.99)	0.85 (0.70-1)

As multivariable methods are known to select different candidate biomarkers, often with equal accuracy [37], we focused on the overlapping miRNAs selected by the four different machine learning methods. From the 20 miRNAs selected across all four methods, seven miRNAs are found in more than one model, of these, two were selected by every model; miR-636 and miR-187-5p (Fig. 2A).

For a subset of patients from Sheffield, NT-proBNP levels were assayed at routine clinical appointments. We then used these to compare the models’ performances when NT-proBNP levels were included (Supplementary Figure 3). Although the best performing miRNA model (RandomForest) did not perform significantly different to the NT-proBNP classifier alone (miRNA AUC 95% CI = 0.69 - 1 vs NT-proBNP AUC 95% CI = 0.84 - 1), all miRNA models with NT-proBNP saw an improved performance with AUCs (Supplementary Figure 3). Random forest increased from 0.84 to 0.97, rpart from 0.79 to 0.81, LASSO increased from 0.78 to 0.93, and the XGBoost model increased from 0.82 to 0.95, though not significantly larger according to the DeLong test. A clear association of miRNAs with PAH diagnosis may warrant future investigation of specific miRNAs for therapeutic intervention.

### 3.3 Importance of individual miRNAs in PAH classification

**Fig. 4.**
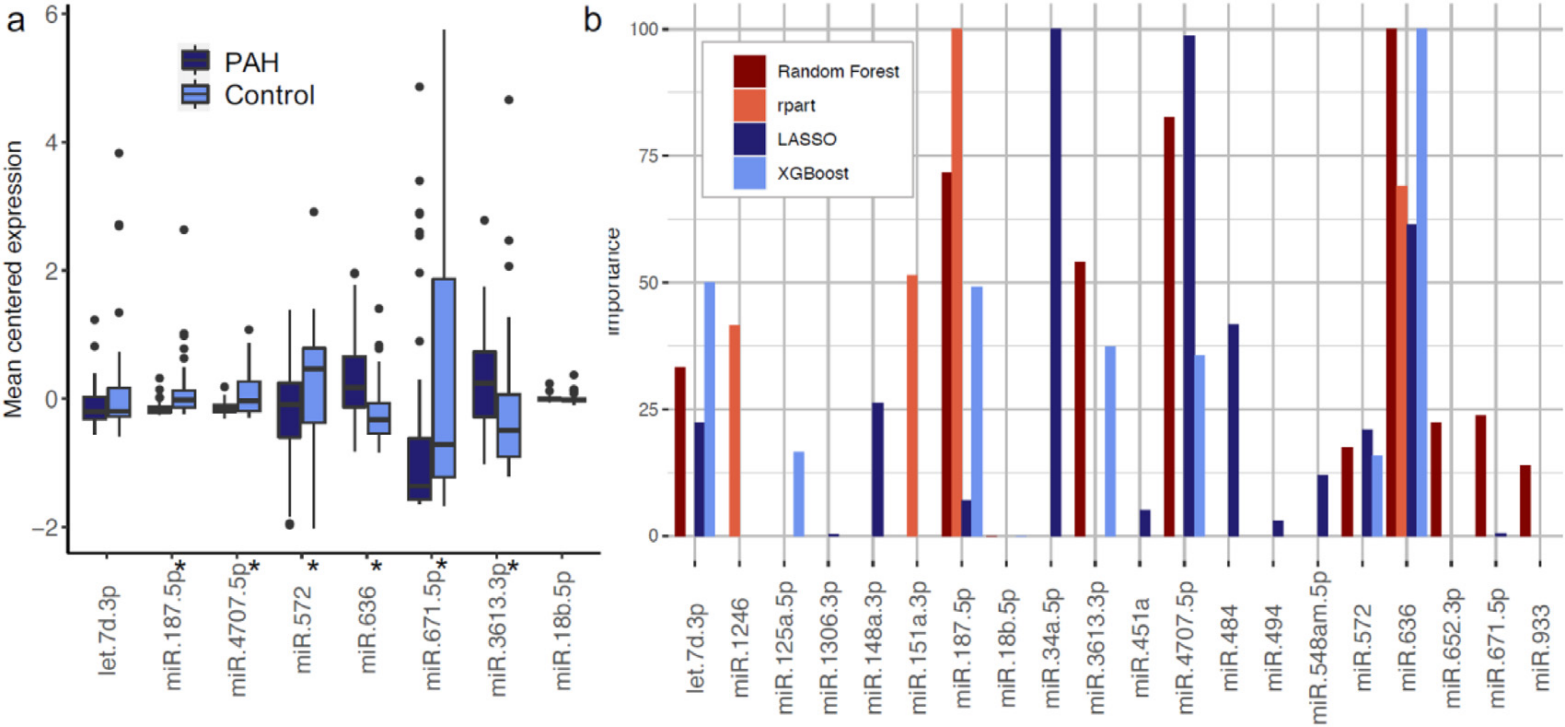
(a) Comparison of mean centered expression values for both training and validation groups (n = 107) of miRNAs for patients with pulmonary arterial hypertension (PAH) and no PH controls (Control) selected by 2 or more feature selection methods. * microRNAs with a significant difference between groups (adjusted p-value for Wilcoxon rank-sum test < 0.05). (b) Variable importance scores for the miRNAs selected by the feature selection methods, scaled between 0 - 100 per method.

To ensure no individual miRNA was driving the classification models, a univariable analysis was carried out (Supplementary Table 8). For each miRNA, the expression levels of patients and controls were compared using a wilcoxon signed-rank test, then controlled for multiple testing using the Benjamini Hochberg correction (38) at 0.05. The mean centered expression values for miRNAs selected by at least two feature selection methods can be seen in Fig. 4a. Ten of the miRNAs identified in the feature selection methods had an adjusted p-value <0.05. We also looked at the univariate discriminatory power of each miRNA individually. MiR-187-5p had an accuracy of 0.78 on the validation set, whereas miR-636 had an accuracy of 0.69. To assess the potential impact of individual miRNAs on disease progression, we also looked at the survival difference in patients when stratifying them based on the median fitted risk of different miRNAs. However, no miRNA had a significant cox proportional hazard p-value (Supplementary Table 9).

### 3.4 PAH classification performs similarly well using miRNA targets

Two miRNAs were identified by all four feature selection methods: miR-187-5p and miR-636. These miRNA were also ranked highest in a variable importance analysis (Fig. 4b). In order to investigate the novel role these miRNAs play in PAH, we predicted their target genes. The two miRNAs had 20 predicted gene targets in common (listed in the supplementary), with 630 targets in total.

Feature selection methods can be unstable when there are few samples for training. To counter this we verified the selected miRNAs gene targets in a previously published whole blood RNA seq data set [34], as well as two independent expression studies [35,36] .

**Fig. 5.**
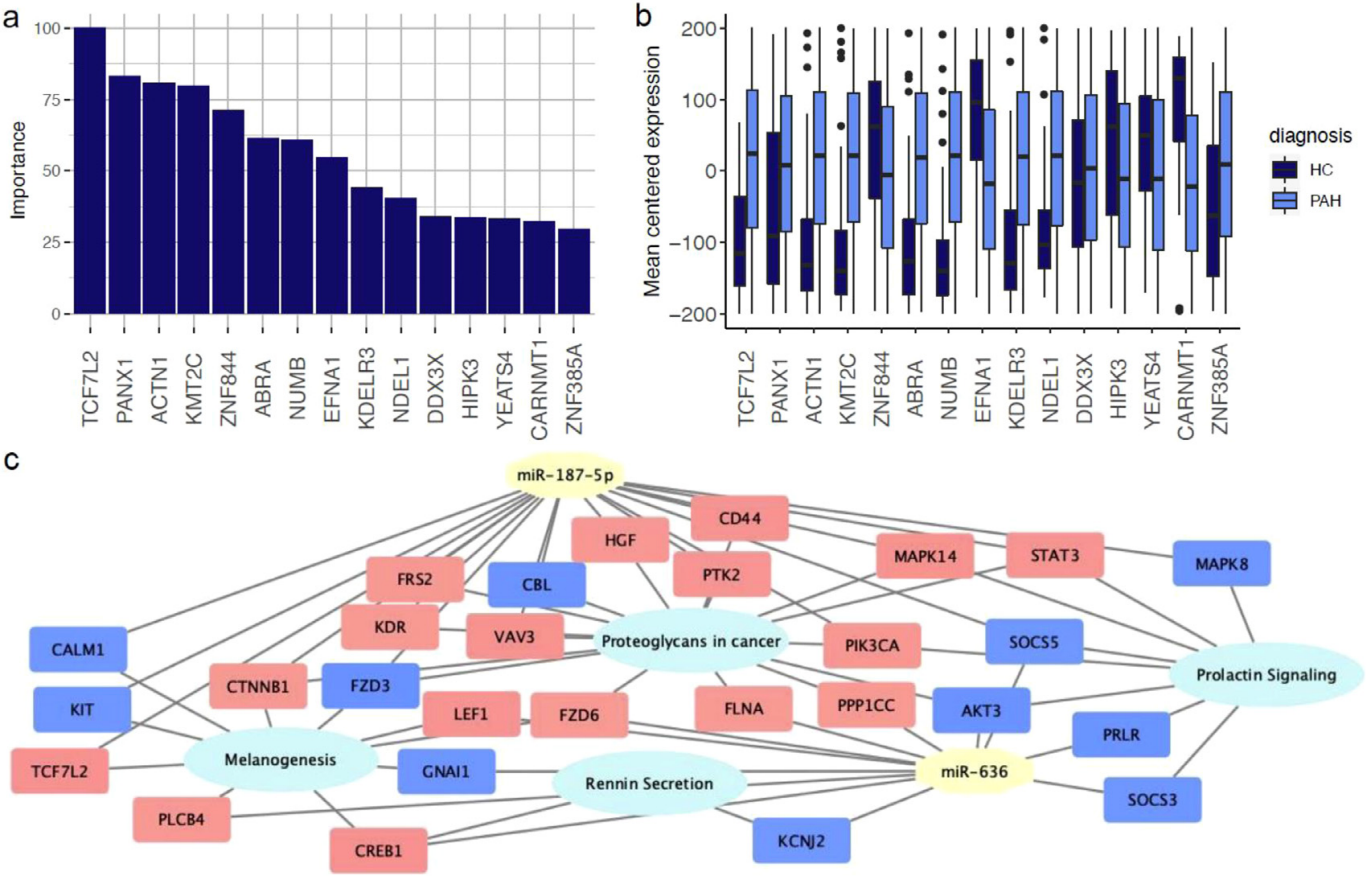
(a) Top 15 genes ranked with the highest importance in classifying patients in an RNAseq dataset (n = 401), scaled between 0 and 100. (b) Mean centered gene expression for top 15 genes (c) Significantly enriched KEGG pathways of the gene targets from miR-636 and miR-187-5p present in the validation RNA seq dataset. Down regulated genes in pink, up-regulated in blue.

The whole blood RNA seq data set contained 54 independent healthy volunteers and 347 PAH patients. Utilising the miRNA target gene set in this RNA seq data set (of which 548 target genes were present), an XGBoost model was used to classify PAH from non-PH, using a cutoff of 0.841. We used XGBoost as a classifier, as the XGBoost model had the highest correct classification rate for the miRNA set. This produced a model with 0.86 AUC (95% CI 0.78-0.94), and an accuracy of 0.89 for the validation set. This classification model also allowed us to rank the genes contributing the most to the model. The top 15 gene targets are shown in Fig. 5.

From the list of 630 target genes, 592 were found in at least one lung tissue dataset. GSE15197 contained 587 of the gene targets, with 281 found to be differentially expressed (adjusted p-value <0.05). All133 predicted gene targets that were profiled in GSE53408 were differentially expressed. Narrowing this down, 61 genes were differentially expressed in the same direction in both datasets. Basic characteristics of the two cohorts are described in Supplementary Table 4. A pathway analysis of all 630 gene targets showed four enriched KEGG pathways: proteoglycans in cancer, rennin secretion, melanogenesis, and prolactin signaling pathway (Fig. 5c). Widening the network to include miRNAs selected by at least two feature selection methods showed that of these miRNAs, miR-3613, miR-671 and miR-18b-5p also targeted genes from all of these pathways, with miR-572 targeting genes in the proteoglycans in cancer pathway.

**Fig. 6.**
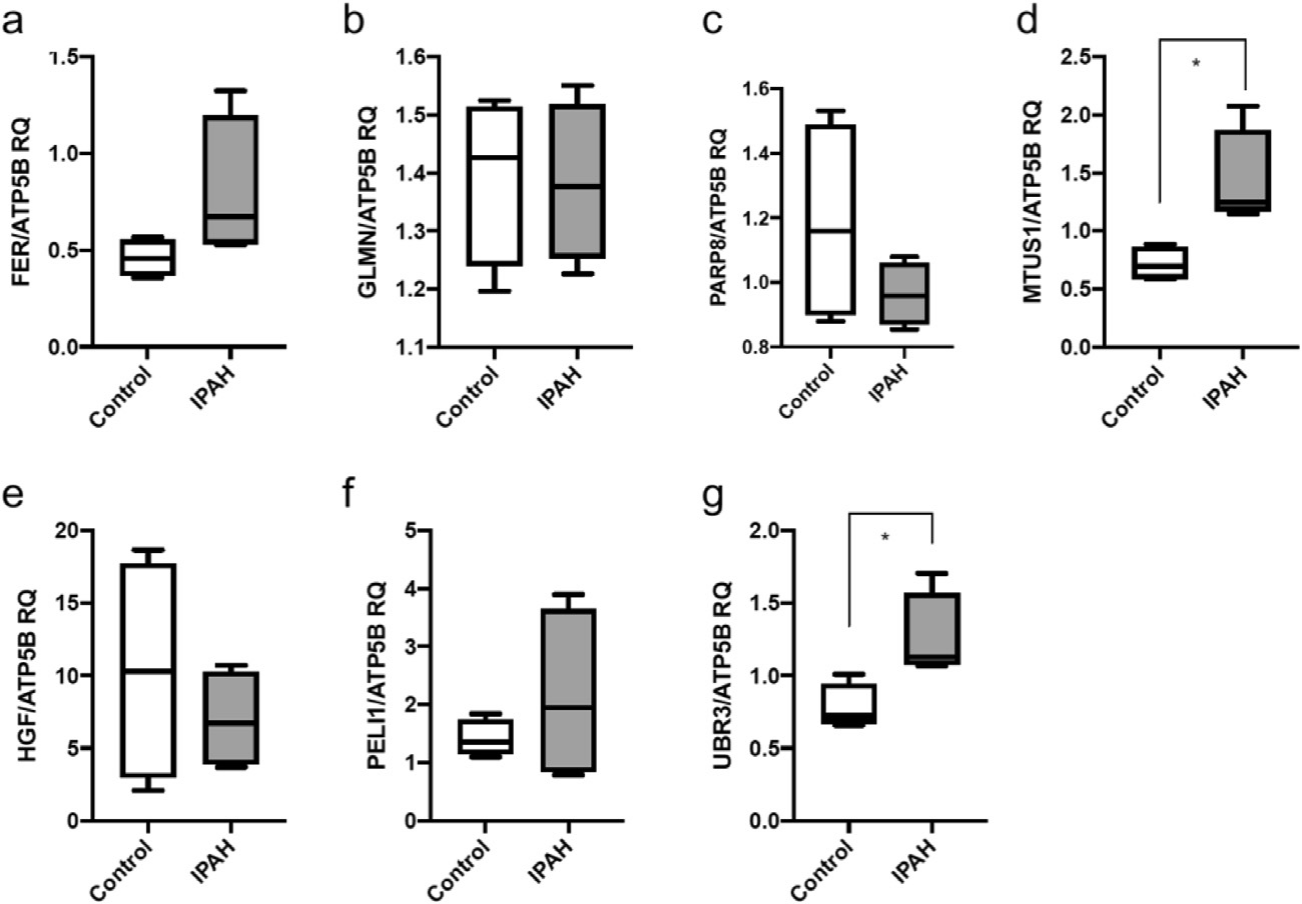
qPCR RQ relative quantification box plots for (a) FER, (b) GLMN, (c) PARP8, (d) MTUS1, (e) HGF, (f) PELI1, (g) UBR3.

From the pathways identified and putative links to PAH pathogenesis, seven gene targets (FER, GLMN, PARP8, MTUS1, HGF, PELI1 and UBR3) were selected for qPCR validation using 4 control human pulmonary artery smooth muscle cells (PASMC) and 4 with IPAH [37]. Two genes in particular, *MTUS1* and *UBR3* showed a significant increase in expression in patient derived PASMCs compared to independent control cells (Fig. 6). There were no significant differences in expression for the other genes.

## 4 Discussion

There is increasing evidence that changes in miRNA expression levels are associated with progression of PAH. Here, we used miRNA expression profiles and a consensus machine learning approach to identify two consistently prioritised miRNAs with high accuracy at identifying PAH from no PH controls, as candidates for further investigation. We subsequently identified putative miRNA gene targets and integrated public lung tissue RNA datasets to validate differential regulation of key miRNA targeted genes, again identifying candidates for further investigation. An extreme gradient boosting method of classifying patients based on the putative gene targets in an overlapping cohort had a similar AUC, providing further validation. This data suggests that combining different approaches for selecting miRNAs can reveal diagnostic biomarkers and insights into regulators of disease.

Of the supervised machine learning approaches we tested, we found that a random forest approach identified patients with PAH with the highest sensitivity, although an XGBoost approach had a similarly high AUC. Adding NT-proBNP to the random forest model resulted in a model with a higher classification accuracy compared to NT-proBNP alone. This shows NT-proBNP and miRNAs may provide complementary phenotypic information and therefore both should be incorporated in future prospective validation analyses.

It is important to consider whether the features selected at each point are true biomarkers or false positives. Machine learning provides an unbiased approach to predicting patient status, but also the potential to identify previously unknown interactions and identify novel biological features [39,40]. Our approach of investigating the biomarkers identified through multiple feature selection techniques increases confidence in the generation of reproducible biomarker panels, and reduces the number of miRNAs for potential clinical investigation. The selected miRNAs ranked highly in terms of variable importance (Fig. 4B).

Both miRNAs selected have previously been linked to PAH. MiR-187 has previously been identified as significantly upregulated in endoarterial biopsy samples in a porcine model [41], and in human lung tissue [42], in concordance with our findings. However, one study on cardiac tissue from the sugen5416 plus hypoxia rat model found miR-187-5p to be significantly downregulated [43]. MiR-636 has been reported to correlate with maximum change in pulmonary vascular resistance (PVR) in a small study on a paediatric PAH population [44]. The above literature reports support the evidence that miR-187-5p and miR-636, identified here as candidate biomarkers may be associated with disease progression of PAH providing validation that our machine learning approach identified miRNA biomarkers of relevance. Several other miRNAs identified as having a high importance score by the feature selection methods have also previously been seen in PAH, for example MiR-4707-5p has been identified as a potential target for PH [45]. Additionally, miR-34 has been seen to have decreased expression in PAH [46,47], and let-7d, which has been identified as a potential biomarker for the presence and severity of PH in patients with SSc [48]. Similarly, the target genes driving the classification in an independent RNAseq dataset, TCF7L2*,* which ranked highest in importance has previously been seen to be differentially expressed in the lung tissue of IPAH patients [49] as well as in the cardiac muscle tissue in a rat model [50] Some of these target genes also showed weak to moderate correlation with available clinical features, such as lung function forced vital capacity (Supplementary Table 10).

Our main aim in this study was to investigate the relationship between miRNAs and clinical classifications, not to develop a diagnostic tool. ML methods can capture more complex, non-linear relationships, where a straightforward univariable analysis cannot. A limitation to this study is the relatively small sample size used to both generate and validate the miRNAs as classifiers. This may have resulted in some model overfitting and therefore a possible overestimation of effect size. In order to mitigate this, we validated the gene targets in separate published datasets, and used qPCR to validate potentially interesting genes. The target gene data contained a far larger number of variables, with 548 genes for each of the 401 subjects, necessitating our use of ML in this dataset. As a result, future studies based on larger retrospective and prospective clinical cohorts are warranted, and currently underway (ClinicalTrials.gov NCT04193046) to corroborate the utility of these, and potentially other miRNAs as classifiers and biomarkers. In such a small cohort, there was a danger the models could have been driven by factors such as age and sex, but classification using only these factors yielded an accuracy of 0.57 in the validation set. We also noted that the AUC confidence intervals for males and females on the training and validation sets overlapped. Additionally, both SSc and PAH, as individual diseases can be heterogeneous [51]. As such within our cohorts of mixed IPAH and SSc-PAH there are likely to be variations between patients, and equally, our control group included 10 disease controls and 33 healthy volunteers. We also attempted a leave one out cross validation approach across the whole dataset, which resulted in similar miRNAs being selected (Supplementary Figure 4). These mixed groups likely reduce the risk of overfitting to a specific patient phenotype, and increase the chance that this analysis could be replicated in other PAH cohorts. The two candidate miRNAs selected from the microarray study have not been further quantified by PCR. However, correlations between miRNA microarray expression and PCR have been shown to have very high correlation coefficients [52]. Consequently, further validation of the two miRNAs identified in a larger, independent cohort are necessary before a clinical application can be considered.

In summary, our approach using four machine learning feature selection algorithms identified a two miR-signature for PAH from patient plasma. These circulating miRNAs, and their target genes may provide a novel PAH signature, reveal novel disease mechanisms and highlight future putative drug targets.

## Contributors

NE and JI helped design and performed experiments and analysis, and helped write the manuscript. JAP helped design and performed experiments, and helped write the manuscript; SK helped with data analysis and helped write the manuscript; CJR & AMKR help prepare the manuscript; RC, CAE, DGK, LSH helped to collect samples, and write the manuscript; JW, AART, NWM & MRW helped design experiments, and helped write the manuscript; DW & AL had the original idea, helped design the experiments and contributed to writing the manuscript. All authors read and approved the final version of the manuscript.

## Declaration of Competing Interest

CJR declares personal consultancy fees from Actelion and United Therapeutics. JW declares personal consultancy fees from Actelion. DK reports grants, personal fees and non-financial support from Acceleron, Janssen, GSK and MSD, outside the submitted work. AART declares non-financial support from Actelion to attend educational events. RC reports personal fees from Janssen Pharmaceuticals, personal fees from MSD, outside the submitted work. LH reports consultancy fees from Bayer, GSK, MSD, and Janssen, outside the submitted work. MRW reports consultancy fees from Novartis, Acceleron, Actelion and MorphogenIX. CAE declares consultancy fees from Janssen-Cilag and Bayer.

AL declares outside of scope and grant funding from Novartis, Janssen, GSK; Conference support from Actelion; Consultancy from Actelion, GSK. All other authors report no conflicts of interest.

## Appendix Supplementary materials

Image, application 1
