# Supplementary material for: A diagnostic miRNA signature for pulmonary arterial hypertension using a consensus machine learning approach
Source: eBioMedicine. 2021 Jun 26;69:103444. doi: 10.1016/j.ebiom.2021.103444 (PMC8243351; doi:10.1016/j.ebiom.2021.103444)
Supplement: Supplementary file 1 [file mmc1.docx]

# **Supplementary Materials**

**Plasma preparation and RNA isolation**

Total RNA was extracted from the plasma using the total RNA slurry format extraction kit (Norgen Biotek Corp. Canada). Prior to experimental start, solution Slurry C2 and Lysis Buffer A were prepared by incubation (60^o^C, 20 minutes). 200 ml of slurry C was added to a 50 ml Falcon tube, then 1800 ml of Lysis Buffer A, 20 ml of 2 M DTT, and 500 ml of plasma were sequentially added. The solution was mixed by vortex (high, 15 seconds) and incubated (60 ^o^C, 10 minutes). 3 ml of 100% ethanol was added and mixed by vortex for 15 seconds then centrifuged (30 seconds, 1000 rpm). Supernatant was discarded and 300 ml of Lysis buffer A added to the slurry pellet, mixed by vortex and incubated (60^o^C, 10 minutes). 300 ml of 100% ethanol was added and the mixture and mixed by vortex for 15 seconds. Up to a maximum of 600 ml of solution was transferred to a mini filter spin column with collection tube and centrifuged (10 000 rpm, 1 minute) and the flow-through discarded. This was repeated with the remaining solution. The column was washed by addition of 400 ml of Wash Solution A and centrifuged (10 000 rpm, 1 minute) the flow-through was discarded. This wash was repeated for a total of 3 washes. The column was dried by centrifugation (14 000 rpm, 3 minutes). The column was transferred to a new DNase/RNase free 1∙5 ml Eppendorf. RNA elution was performed by addition of 100 ml of Elution Solution A and centrifugation (2 000 rpm, 2 minutes followed by 14 000 rpm, 3 minutes). The column was discarded and RNA stored at -80^o^C.

**Overlapping putative gene targets of miR-636 & miR-187-5p:**

VAMP7, LMO3, DGKH, YTHDF3, DNAL1, PPP2R2A, ZDHHC15, UBN2, CDKN1B, FAM63B, PARP15, SOCS5, ZNF844, HECTD2, RIMS3, ZNF720, FCHO2, CBX5, PALM2, GABRB2


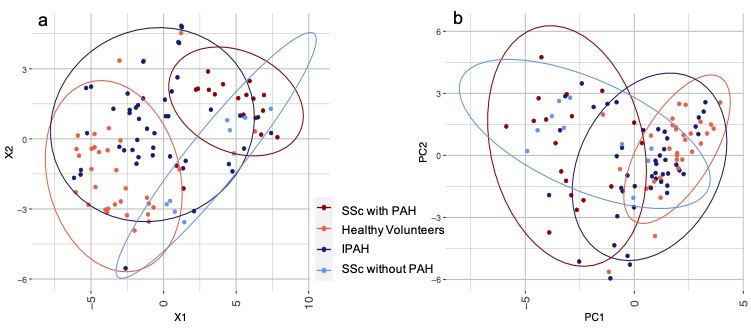


**Supplementary Figure 1**: a) t-SNE plot of subjects in both the training and validation sets. b) PCA plot of subjects in both the training and validation sets, showing the first 2 principal components. PC1 (27∙1% of variance), and PC2 (21∙4% of variance).


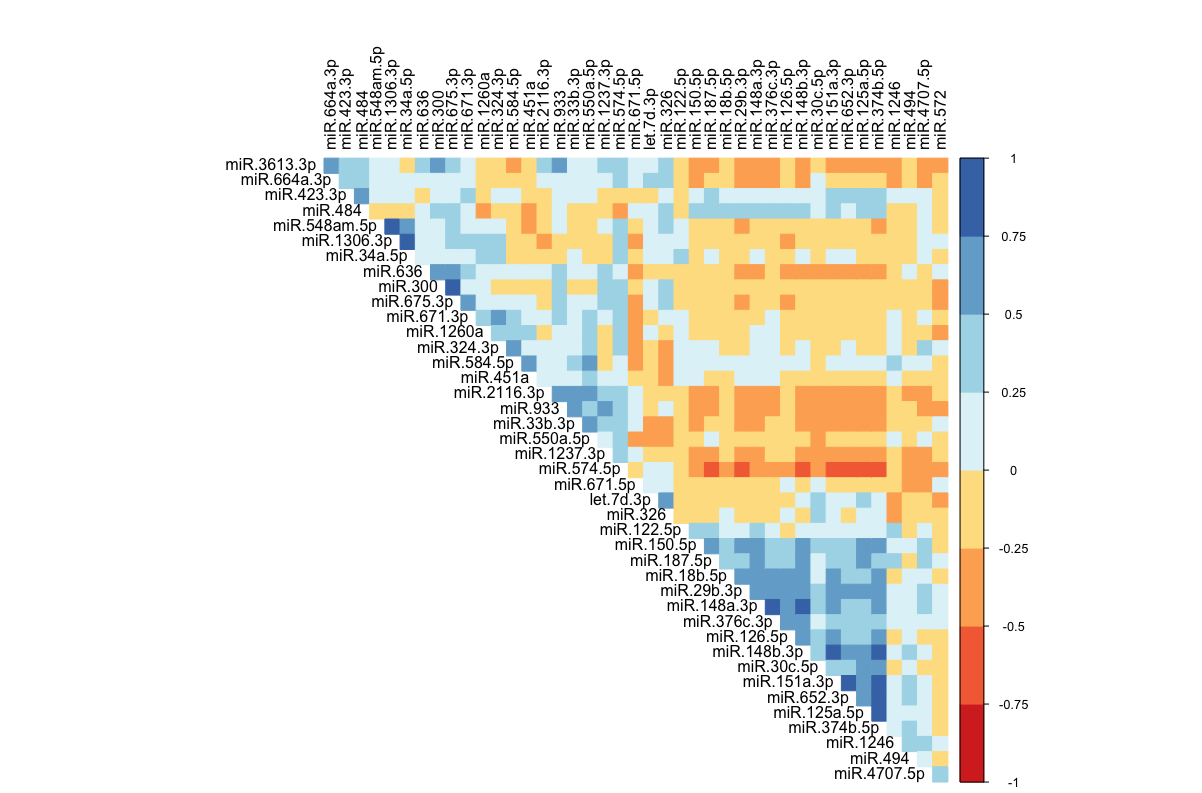
**Supplementary Figure 2**: Correlation plot of the miRNAs remaining after filtering out those with high correlation (Spearman’s > 0∙7)


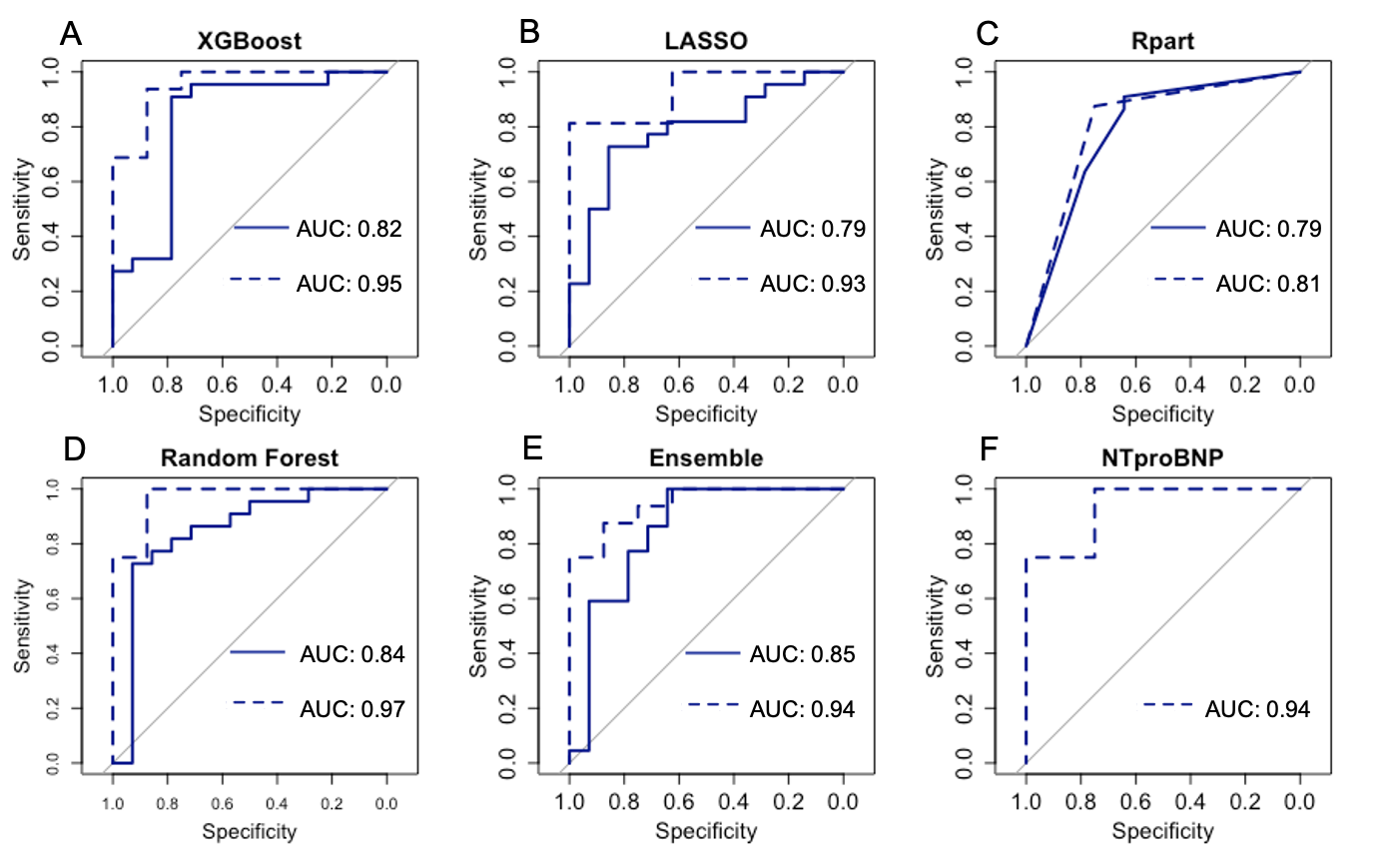


**Supplementary Figure 3**: Solid lines indicate miRNA models, dashed lines indicate miRNA model + NTproBNP. ROC curves for all 4 machine learning classifiers on the validation set, and NTproBNP. (a) extreme gradient boosting (XGBoost) utilising 8 miRNAs; (b) LASSO utilising 13 miRNAs; (c) regression partition trees (Rpart) utilising 4 miRNAs; (d) a random forest wrapper method (boruta) utilising 10 miRNAs; (e) Ensemble approach utilising 20 miRNAs; (f) NTproBNP alone.


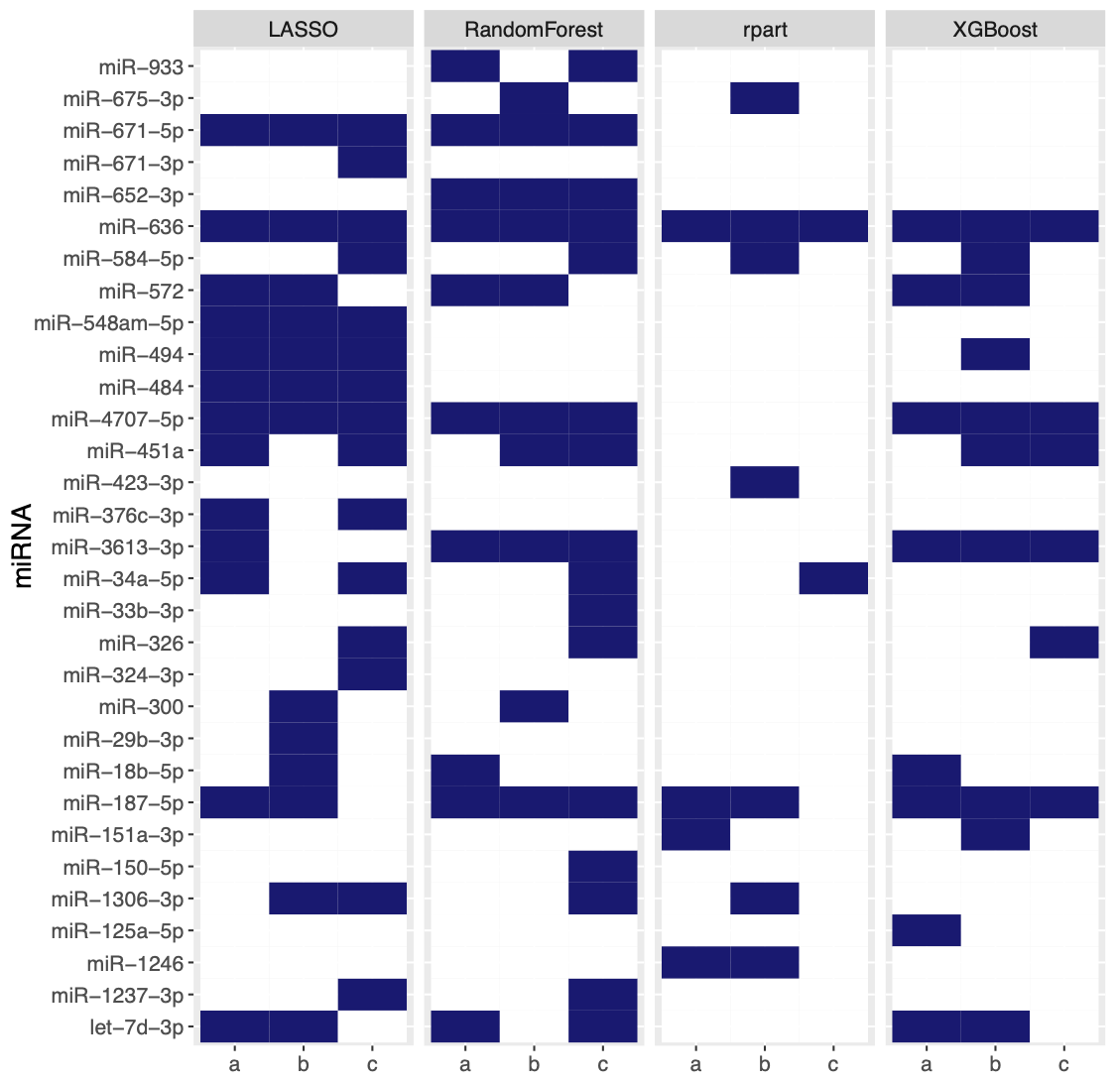


**Supplementary Figure 4:** Heatmap of selected miRNAs using 4 different supervised machine learning approaches across 3 different discovery sets: a) a training and validation cross validation approach for IPAH and PAH-SSc vs healthy controls and PH-without SSc; b) leave-one-out cross validation approach across the whole dataset for IPAH and PAH-SSc vs healthy controls and PH-without SSc. c) training and validation cross validation approach for patients with IPAH and healthy controls. Blue: miRNA was selected, white: miRNA was not selected

**Supplementary Table 1:** Missing values for PAH patient’s data (Table 1)

| Parameter | No missing training data (%) | No missing validation data (%) | Total no missing (%) |
| --- | --- | --- | --- |
| Age | 0 | 0 | 0 |
| Sex | 0 | 0 | 0 |
| Alive at 5 year follow up | 0 | 0 | 0 |
| WHO functional class | 0 | 0 | 0 |
| Patients on immunomodulatory agent at sampling | 0 | 0 | 0 |
| Mean Pulmonary Arterial Pressure | 2 (5) | 3 (14) | 5 (8) |
| Pulmonary vascular resistance | 6 (14) | 4 (19) | 10 (16) |
| 6 minute walk distance (Imperial only) | 2 (25) | 2 (50) | 4 (33) |
| ISWD (Sheffield only) | 7 (20) | 2 (12) | 9 (17) |
| Cardiac Output (l/min) | 3 (7) | 4 (19) | 7 (11) |
| Mean capillary wedge pressure | 5 (12) | 5 (24) | 10 (16) |

**Supplementary Table 2:** Tripod Checklist

| Section/Topic | Item |  | Checklist Item | Section |
| --- | --- | --- | --- | --- |
| Title and abstract | | | | |
| Title | 1 | D;V | Identify the study as developing and/or validating a multivariable prediction model, the target population, and the outcome to be predicted. | Title |
| Abstract | 2 | D;V | Provide a summary of objectives, study design, setting, participants, sample size, predictors, outcome, statistical analysis, results, and conclusions. | Abstr. |
| Introduction | | | | |
| Background and objectives | 3a | D;V | Explain the medical context (including whether diagnostic or prognostic) and rationale for developing or validating the multivariable prediction model, including references to existing models. | Intro |
|  | 3b | D;V | Specify the objectives, including whether the study describes the development or validation of the model or both. | Intro |
| Methods | | | | |
| Source of data | 4a | D;V | Describe the study design or source of data (e.g., randomized trial, cohort, or registry data), separately for the development and validation data sets, if applicable. | 2.1 |
|  | 4b | D;V | Specify the key study dates, including start of accrual; end of accrual; and, if applicable, end of follow-up. | 2.1 |
| Participants | 5a | D;V | Specify key elements of the study setting (e.g., primary care, secondary care, general population) including number and location of centres. | 2.1 |
|  | 5b | D;V | Describe eligibility criteria for participants. | 2.1 |
|  | 5c | D;V | Give details of treatments received, if relevant. | 2.1, Table 1 |
| Outcome | 6a | D;V | Clearly define the outcome that is predicted by the prediction model, including how and when assessed. | 2.2.1 |
|  | 6b | D;V | Report any actions to blind assessment of the outcome to be predicted. | n/a |
| Predictors | 7a | D;V | Clearly define all predictors used in developing or validating the multivariable prediction model, including how and when they were measured. | 2.1.2 |
|  | 7b | D;V | Report any actions to blind assessment of predictors for the outcome and other predictors. | n/a |
| Sample size | 8 | D;V | Explain how the study size was arrived at. | 2.1 |
| Missing data | 9 | D;V | Describe how missing data were handled (e.g., complete-case analysis, single imputation, multiple imputation) with details of any imputation method. | 2.1.2, Suppl Table 1 |
| Statistical analysis methods | 10a | D | Describe how predictors were handled in the analyses. | 2.2 |
|  | 10b | D | Specify type of model, all model-building procedures (including any predictor selection), and method for internal validation. | 2.2.1 |
|  | 10c | V | For validation, describe how the predictions were calculated. | 2.2.3 |
|  | 10d | D;V | Specify all measures used to assess model performance and, if relevant, to compare multiple models. | 2.2.3 |
|  | 10e | V | Describe any model updating (e.g., recalibration) arising from the validation, if done. | n/a |
| Risk groups | 11 | D;V | Provide details on how risk groups were created, if done. | n/a |
| Development vs. validation | 12 | V | For validation, identify any differences from the development data in setting, eligibility criteria, outcome, and predictors. | 2.4 & 2.5 |
| Results | | | | |
| Participants | 13a | D;V | Describe the flow of participants through the study, including the number of participants with and without the outcome and, if applicable, a summary of the follow-up time. A diagram may be helpful. | 2.1 & Tab 1 |
|  | 13b | D;V | Describe the characteristics of the participants (basic demographics, clinical features, available predictors), including the number of participants with missing data for predictors and outcome. | Table 1 & Suppl Tab 1 |
|  | 13c | V | For validation, show a comparison with the development data of the distribution of important variables (demographics, predictors and outcome). | Table 1 |
| Model development | 14a | D | Specify the number of participants and outcome events in each analysis. | Table 1 |
|  | 14b | D | If done, report the unadjusted association between each candidate predictor and outcome. | n/a |
| Model specification | 15a | D | Present the full prediction model to allow predictions for individuals (i.e., all regression coefficients, and model intercept or baseline survival at a given time point). | 2.2 & Suppl Tabs 3 & 4 |
|  | 15b | D | Explain how to use the prediction model. | 2.2.1 |
| Model performance | 16 | D;V | Report performance measures (with CIs) for the prediction model. | Table 2 |
| Model- updating | 17 | V | If done, report the results from any model updating (i.e., model specification, model performance). | n/a |
| Discussion | | | | |
| Limitations | 18 | D;V | Discuss any limitations of the study (such as nonrepresentative sample, few events per predictor, missing data). | 4 |
| Interpretation | 19a | V | For validation, discuss the results with reference to performance in the development data, and any other validation data. | 4 |
|  | 19b | D;V | Give an overall interpretation of the results, considering objectives, limitations, results from similar studies, and other relevant evidence. | 4 |
| Implications | 20 | D;V | Discuss the potential clinical use of the model and implications for future research. | 4 |
| Other information | | | | |
| Supplementary information | 21 | D;V | Provide information about the availability of supplementary resources, such as study protocol, Web calculator, and data sets. | 8 |
| Funding | 22 | D;V | Give the source of funding and the role of the funders for the present study. | 2.7 |

**Supplementary Table 3**: Parameters used to optimise an XGBoost classifier for PAH using miRNAs. a: the range of each parameter tuned, b: the optimal parameter for the initial xgboost model, c: the final parameter value used for an xgboost model trained on a reduced number of miRNAs.

| **Parameter** | **Available Range** | **Optimisation range**^a^ | **Initial value**^b^ | **Optimal value**^c^ |
| --- | --- | --- | --- | --- |
| No of trees | 1 - ∞ | 100 - 10 000 | 4300 | 200 |
| Learning rate | 0 - 1 | 0∙01, 0∙025, 0∙05, 0∙1, 0∙2, 0∙3 | 0∙025 | 0∙025 |
| Maximum tree depth | 0 - ∞ | 1, 2, 3, 4, 5, 6 | 1 | 1 |
| gamma | 0 - ∞ | 0, 0∙05, 0∙1, 0∙5, 0∙7, 0∙9, 1 | 0∙05 | 1 |
| Minimum child weight | 0 - ∞ | 1,2,3,4 | 2 | 1 |
| Subsample rate (row sampling) | 0 - 1 | 0∙5, 0∙75, 1∙0 | 0∙5 | 0∙5 |
| % feature used in each boost (column sampling) | 0 - 1 | 0∙4, 0∙6, 0∙8, 1∙0 | 0∙4 | 0.4 |

**Supplementary Table 4**: Parameters used to optimise an XGBoost classifier for PAH using mRNAs. a: the range of each parameter tuned, b: the optimal parameter for the xgboost model

| **Parameter** | **Available Range** | **Optimisation range**^a^ | **Final value**^b^ |
| --- | --- | --- | --- |
| No of trees | 1 - ∞ | 100 - 10 000 | 550 |
| Learning rate | 0 - 1 | 0∙01, 0∙025, 0∙05, 0∙1, 0∙2, 0∙3 | 0∙05 |
| Maximum tree depth | 0 - ∞ | 1, 2, 3, 4, 5, 6 | 3 |
| gamma | 0 - ∞ | 0, 0∙05, 0∙1, 0∙5, 0∙7, 0∙9, 1 | 0.05 |
| Minimum child weight | 0 - ∞ | 0∙2, 0∙5, 1, 2 | 0∙2 |
| Subsample rate (row sampling) | 0 - 1 | 0∙5, 0∙75, 1∙0 | 0∙5 |
| % feature used in each boost (column sampling) | 0 - 1 | 0∙4, 0∙6, 0∙8, 1∙0 | 0∙4 |

**Supplementary Table 5:** Characteristics of 2 GEO datasets, GSE15197 and GSE53408. *Information missing for 4 patients and 3 controls

| **GSE15197** | ***n*** | **Age, yr** | **Sex (M/F)** | **PVRI, Wood units** | **MPAP, mmHg** |
| --- | --- | --- | --- | --- | --- |
| GSE15197 PAH | 18 | 44 ± 10 | 7/11 | 20 ± 9 | 55 ± 7 |
| GSE15197 Normal controls | 13 | 60 ± 11 | 5/8 |  |  |
| GSE53408 PAH* | 8 | 40 ± 12 | 3/5 |  | 56 ± 9 |
| GSE53408 Normal controls* | 8 | 47 ± 15 | 4/4 |  |  |

**Supplementary Table 6**: Model Classifications on the validation set for four different methods; regression partition trees (Rpart), LASSO, random forest wrapper (Boruta), extreme gradient boosting (XGBoost) and an ensemble prediction

| **Patient ID** | **Diagnosis** | **Random Forest prediction** | **Rpart prediction** | **LASSO prediction** | **XGBoost prediction** | **Ensemble prediction** |
| --- | --- | --- | --- | --- | --- | --- |
| 1 | Healthy volunteer | PAH | PAH | PAH | Control | PAH |
| 2 | Healthy volunteer | Control | Control | Control | Control | Control |
| 3 | Healthy volunteer | Control | Control | Control | Control | Control |
| 4 | Healthy volunteer | Control | Control | Control | Control | Control |
| 5 | Healthy volunteer | Control | Control | Control | Control | Control |
| 6 | Healthy volunteer | Control | Control | Control | Control | Control |
| 7 | Healthy volunteer | Control | Control | Control | Control | Control |
| 8 | Healthy volunteer | Control | Control | PAH | Control | Control |
| 9 | Healthy volunteer | Control | PAH | Control | Control | PAH |
| 10 | Healthy volunteer | PAH | PAH | PAH | PAH | PAH |
| 11 | Healthy volunteer | Control | Control | Control | PAH | Control |
| 12 | SSc-without PAH | PAH | PAH | PAH | PAH | PAH |
| 13 | SSc-without PAH | PAH | PAH | PAH | PAH | PAH |
| 14 | SSc-without PAH | Control | Control | Control | Control | Control |
| 15 | SSc-PAH | Control | PAH | PAH | Control | PAH |
| 16 | SSc-PAH | PAH | PAH | PAH | PAH | PAH |
| 17 | SSc-PAH | PAH | PAH | PAH | PAH | PAH |
| 18 | SSc-PAH | PAH | PAH | Control | PAH | PAH |
| 19 | SSc-PAH | PAH | PAH | PAH | PAH | PAH |
| 20 | SSc-PAH | PAH | PAH | PAH | PAH | PAH |
| 21 | SSc-PAH | PAH | PAH | PAH | PAH | PAH |
| 22 | IPAH | PAH | PAH | PAH | PAH | PAH |
| 23 | IPAH | PAH | PAH | PAH | PAH | PAH |
| 24 | IPAH | PAH | PAH | PAH | PAH | PAH |
| 25 | IPAH | PAH | PAH | PAH | PAH | PAH |
| 26 | IPAH | PAH | PAH | PAH | PAH | PAH |
| 27 | IPAH | PAH | PAH | PAH | PAH | PAH |
| 28 | IPAH | PAH | PAH | PAH | PAH | PAH |
| 29 | IPAH | PAH | Control | PAH | PAH | PAH |
| 30 | IPAH | PAH | PAH | PAH | PAH | PAH |
| 31 | IPAH | PAH | PAH | PAH | PAH | PAH |
| 32 | IPAH | PAH | PAH | Control | PAH | PAH |
| 33 | IPAH | PAH | Control | Control | PAH | Control |
| 34 | IPAH | PAH | PAH | PAH | PAH | PAH |
| 35 | IPAH | Control | PAH | Control | Control | Control |
| 36 | IPAH | Control | PAH | Control | PAH | PAH |

**Supplementary Table 7**: Mean 10 fold cross-validated performance on the training set regression partition trees (Rpart), a random forest wrapper method (boruta), LASSO, and extreme gradient boosting (XGBoost).

|  | **Random forest** | **Rpart** | **LASSO** | **XGBoost** |
| --- | --- | --- | --- | --- |
| Sensitivity | 0∙72 | 0∙50 | 0∙65 | 0∙75 |
| Specificity | 0∙93 | 0∙64 | 0∙83 | 0∙88 |
| Positive predictive value | 0∙91 | 0∙53 | 0∙79 | 0∙85 |
| Negative predictive value | 0∙85 | 0∙66 | 0∙79 | 0∙86 |
| Correct classification rate | 0∙85 | 0∙58 | 0∙76 | 0∙83 |

**Supplementary Table 8**: Minimum, mean and maximum values for 43 miRNAs remaining when correlating miRNAs have been filtered out for the validation set, grouped by patients with pulmonary arterial hypertension, and healthy and disease controls.

| **miRNA** | **PAH patients** | | | **Healthy and disease controls** | | |
| --- | --- | --- | --- | --- | --- | --- |
|  | **Min** | **Mean** | **Max** | **Min** | **Mean** | **Max** |
| let-7d-3p | 2∙726 | 3∙442 | 7∙135 | 2∙752 | 3∙132 | 4∙537 |
| miR-122-5p | 2∙618 | 3∙064 | 8∙362 | 2∙597 | 2∙888 | 3∙971 |
| miR-1237-3p | 2∙674 | 3∙472 | 4∙685 | 3∙106 | 3∙779 | 4∙533 |
| miR-1246 | 2∙595 | 3∙909 | 6∙123 | 2∙649 | 4∙067 | 13∙529 |
| miR-125a-5p | 2∙608 | 2∙876 | 5∙046 | 2∙626 | 2∙714 | 3∙657 |
| miR-126-5p | 2∙654 | 2∙956 | 3∙686 | 2∙713 | 2∙911 | 3∙354 |
| miR-1260a | 5∙628 | 7∙489 | 9∙274 | 5∙949 | 7∙532 | 8∙466 |
| miR-1306-3p | 2∙585 | 2∙976 | 4∙329 | 2∙581 | 2∙806 | 3∙545 |
| miR-148a-3p | 2∙581 | 2∙829 | 3∙771 | 2∙612 | 2∙691 | 2∙998 |
| miR-148b-3p | 2∙589 | 2∙782 | 3∙579 | 2∙585 | 2∙69 | 3∙143 |
| miR-150-5p | 2∙62 | 3∙325 | 7∙367 | 2∙646 | 2∙972 | 5∙987 |
| miR-151a-3p | 2∙577 | 2∙806 | 4∙406 | 2∙593 | 2∙657 | 3∙215 |
| miR-187-5p | 2∙473 | 2∙822 | 5∙34 | 2∙453 | 2∙558 | 3∙024 |
| miR-18b-5p | 2∙576 | 2∙635 | 2∙787 | 2∙586 | 2∙631 | 2∙684 |
| miR-2116-3p | 2∙758 | 3∙121 | 4∙053 | 2∙852 | 3∙255 | 4∙597 |
| miR-29b-3p | 2∙639 | 2∙812 | 3∙285 | 2∙638 | 2∙773 | 2∙944 |
| miR-300 | 2∙65 | 2∙896 | 3∙557 | 2∙75 | 3∙157 | 5∙633 |
| miR-30c-5p | 2∙611 | 2∙952 | 3∙691 | 2∙647 | 2∙873 | 3∙634 |
| miR-324-3p | 2∙931 | 3∙712 | 4∙997 | 2∙816 | 3∙517 | 4∙56 |
| miR-326 | 2∙624 | 2∙809 | 3∙209 | 2∙654 | 2∙781 | 3∙353 |
| miR-33b-3p | 2∙728 | 3∙169 | 4∙454 | 2∙898 | 3∙324 | 4∙471 |
| miR-34a-5p | 2∙682 | 2∙892 | 3∙478 | 2∙649 | 2∙798 | 3∙087 |
| miR-3613-3p | 2∙882 | 3∙881 | 8∙708 | 3∙455 | 4∙394 | 6∙831 |
| miR-374b-5p | 2∙545 | 2∙769 | 3∙496 | 2∙6 | 2∙701 | 3∙565 |
| miR-376c-3p | 2∙524 | 2∙707 | 3∙811 | 2∙546 | 2∙611 | 2∙762 |
| miR-423-3p | 2∙52 | 2∙622 | 3∙06 | 2∙531 | 2∙601 | 2∙782 |
| miR-451a | 6∙925 | 10∙525 | 14∙237 | 7∙365 | 11∙645 | 14∙218 |
| miR-4707-5p | 2∙499 | 2∙862 | 3∙648 | 2∙482 | 2∙638 | 2∙836 |
| miR-484 | 2∙707 | 3∙117 | 3∙821 | 2∙765 | 3∙067 | 3∙849 |
| miR-494 | 2∙642 | 3∙961 | 8∙154 | 2∙637 | 4∙426 | 8∙353 |
| miR-548am-5p | 2∙688 | 2∙997 | 4∙141 | 2∙701 | 2∙908 | 3∙563 |
| miR-550a-5p | 2∙567 | 2∙804 | 3∙454 | 2∙66 | 2∙861 | 3∙473 |
| miR-572 | 2∙541 | 4∙645 | 7∙474 | 2∙62 | 4∙203 | 5∙936 |
| miR-574-5p | 3∙761 | 7∙854 | 11∙055 | 6∙992 | 8∙464 | 10∙159 |
| miR-584-5p | 2∙606 | 3∙487 | 4∙924 | 2∙66 | 3∙839 | 5∙977 |
| miR-636 | 2∙721 | 3∙269 | 4∙954 | 2∙737 | 3∙95 | 5∙509 |
| miR-652-3p | 2∙498 | 2∙627 | 3∙596 | 2∙506 | 2∙543 | 2∙737 |
| miR-664a-3p | 2∙759 | 3∙283 | 5∙719 | 2∙869 | 3∙294 | 3∙693 |
| miR-671-3p | 2∙607 | 2∙684 | 2∙85 | 2∙593 | 2∙692 | 3∙01 |
| miR-671-5p | 2∙492 | 4∙682 | 9∙917 | 2∙538 | 3∙699 | 9∙023 |
| miR-675-3p | 2∙642 | 3∙1 | 4∙601 | 2∙722 | 3∙417 | 6∙002 |
| miR-933 | 2∙601 | 2∙983 | 3∙67 | 2∙796 | 3∙143 | 3∙8 |

**Supplementary Table 9**: Cox proportional hazard for miRNAs selected by a feature selection method∙

| **microRNA** | **beta** | **Wald test** | **P value for miRNA** | **HR (95% CI for HR)** |
| --- | --- | --- | --- | --- |
| hsa-let-7d-3p | -0∙0691 | 0∙05 | 0∙816 | 0∙933 (0∙521-1∙67) |
| hsa-miR-1306-3p | 0∙755 | 3∙17 | 0∙0749 | 2∙13 (0∙927-4∙88) |
| hsa-miR-148a-3p | -0∙4 | 0∙21 | 0∙647 | 0∙671 (0∙121-3∙71) |
| hsa-miR-187-5p | -0∙37 | 0∙39 | 0∙535 | 0∙691 (0∙215-2∙22) |
| hsa-miR-34a-5p | 1∙3 | 2∙2 | 0∙138 | 3∙67 (0∙657-20∙5) |
| hsa-miR-451a | -0∙0776 | 0∙35 | 0∙556 | 0∙925 (0∙715-1∙2) |
| hsa-miR-4707-5p | 0∙257 | 0∙21 | 0∙646 | 1∙29 (0∙432-3∙87) |
| hsa-miR-484 | -1∙53 | 2∙16 | 0∙142 | 0∙216 (0∙028-1∙67) |
| hsa-miR-494 | 0∙0832 | 0∙26 | 0∙608 | 1∙09 (0∙791-1∙49) |
| hsa-miR-548am-5p | 0∙397 | 0∙4 | 0∙525 | 1∙49 (0∙437-5∙06) |
| hsa-miR-572 | 0∙167 | 0∙57 | 0∙451 | 1∙18 (0∙766-1∙82) |
| hsa-miR-636 | 0∙218 | 0∙27 | 0∙6 | 1∙24 (0∙551-2∙81) |
| hsa-miR-671-5p | -0∙0273 | 0∙08 | 0∙782 | 0∙973 (0∙802-1∙18) |
| hsa-miR-18b-5p | -0∙0098 | 0 | 0∙998 | 0∙99 (0∙000646-1520) |
| hsa-miR-3613-3p | -0∙365 | 1∙67 | 0∙196 | 0∙694 (0∙399-1∙21) |
| hsa-miR-652-3p | -0∙431 | 0∙21 | 0∙65 | 0∙65 (0∙101-4∙18) |
| hsa-miR-933 | -1∙48 | 1∙97 | 0∙16 | 0∙228 (0∙029-1∙79) |
| hsa-miR-151a-3p | -0∙493 | 0∙57 | 0∙45 | 0∙611 (0∙17-2∙2) |
| hsa-miR-1246 | 0∙266 | 1∙53 | 0∙216 | 1∙31 (0∙856-1∙99) |
| hsa-miR-125a-5p | -0∙25 | 0∙21 | 0∙65 | 0∙778 (0∙264-2∙3) |

**Supplementary Table 10:** Spearman’s correlation coefficients for gene targets with sample demographics. Highest correlation coefficient reported.

| Clinical Variables | Gene | Absolute max correlation coefficient |
| --- | --- | --- |
| Demographics |  |  |
| Age at Sampling | EFNA1 | -0.3044634 |
| Survival |  |  |
| Survival (years since sampling). | BRWD1 | -0.2952542 |
| Died during Follow up | KAT2A | -0.2616826 |
| HLA-DPA1/DPB1 SNP (rs28568300) | MSI2 | -0.228825 |
| Lung Function | KDM6A | -0.4217336 |
| Forced Vital Capacity (FVC, L) |  |  |
| Forced Expiratory Volume in 1 second (FEV, L) | KDM6A | -0.3891385 |
| Carbon monoxide transfer (KCO %Pred) | NET1 | 0.2068199 |
| Right Heart Catheter |  |  |
| Mean Right Atrial Pressure (mm Hg) | HDGF | 0.2827278 |
| Pulmonary Arterial Wedge Pressure | NDRG4 | -0.2265114 |
| Pulmonary Vascular Resistance | BTBD3 | -0.2398163 |
| Cardiac Index | BTBD3 | 0.2597571 |
| Exercise Capacity |  |  |
| 6 minute walk distance (6MWD,m) | HDGF | -0.2896662 |
| Incremental Shuttle Walk Distance (ISWD,m) | HDGF | -0.2885012 |
